# Supplementary material for: Confocal interferometric scattering microscopy reveals 3D nanoscopic structure and dynamics in live cells
Source: Nat Commun. 2023 Apr 7;14:1962. doi: 10.1038/s41467-023-37497-7 (PMC10081331; doi:10.1038/s41467-023-37497-7)
Supplement: Supplementary file 1 — Supplementary Information [file 41467_2023_37497_MOESM1_ESM.pdf]

Confocal Interferometric Scattering Microscopy Reveals 3D Nanoscopic Structure and Dynamics in Live Cells. Supplementary Information

Michelle Küppers[1,2,3], David Albrecht[1,2], Anna D. Kashkanova[1,2], Jennifer Lühr[1,2] and Vahid Sandoghdar[*,1,2,3]

[1]Max Planck Institute for the Science of Light, 91058 Erlangen, Germany
[2]Max-Planck-Zentrum für Physik und Medizin, 91058 Erlangen, Germany
[3]Friedrich-Alexander-Universität Erlangen-Nürnberg, 91058 Erlangen, Germany
[*] vahid.sandoghdar@mpl.mpg.de

Contents

1 Characterization of the reference electric field **2**

2 Background correction **3**

3 PSF characterization **4**

4 Spherical aberration **5**

5 Scan speed **5**

6 Multiplane reconstruction workflow **6**
6.1 Extracting a ridge . . . 7
6.2 Fitting a model function based on the C-iSCAT PSF for sub-pixel localization . . . 7

7 Mapping a model interface **8**
7.1 Movable cleaved fiber end . . . 8
7.2 Nanofabricated staircase sample . . . 9

8 Mapping the nuclear envelope **12**

9 Multiplane reconstruction of the endoplasmic reticulum **16**

10 Training of the cGAN neural network for segmentation **18**

11 External reference for depth extension **19**

12 Phototoxicity **21**

Supplementary Note 1: Characterization of the reference electric field

The iSCAT signal is composed of the product of the scattered light with a reference. In the simplest arrangement, the reference is obtained from the reflection at the sample interface. In this case, the transmission of the reference field through the pinhole is changed as one moves the focus of the microscope objective for axial scans. To characterize the intensity of the reference electric field in confocal iSCAT measurements, we imaged the reflection at a glass-water interface at different focal plane positions. We set the pinhole width in the detection path to the smallest used in the experiment, namely, to 0.3AU, where one airy unit (AU) is defined as $1\text{AU} = 1.22\lambda/\text{NA}$. Supplementary Figure 1a shows the measured profiles for an incident illumination wavelength of $\lambda = 445$ nm and an oil-immersion objective with NA=1.46. The intensity of the reflected light strongly decreases after about 2 µm and remains measurable for up to about 4 µm above the cover glass (see inset). We note that the variations of the reference field are eliminated because the iSCAT contrast value for each focal plane is computed by subtracting and then dividing the detected intensity to the reference intensity. Furthermore, we note that given the quadratic relation between field and intensity, the drop in the reflected field is much less severe than that of the intensity (compare Supplementary Figs. 1a and 1b). These measurements were performed at a laser power of 5 µW, which was the maximum power used in our experiment for measurements at large depths, e.g., the apical nuclear envelope.

Next, we studied the reference intensity as a function of the focus position for various sizes of the pinhole, ranging from a width of 0.3AU to 1.3AU. For these measurements, we used a laser power of 3 µW and kept the settings of the PMT gain and laser power constant over z and for all pinhole settings. It is clear that the signal drops for smaller pinholes. Supplementary Figure 1c plots the detected power as a function of the pinhole size. As expected, we observe a quadratic behavior.

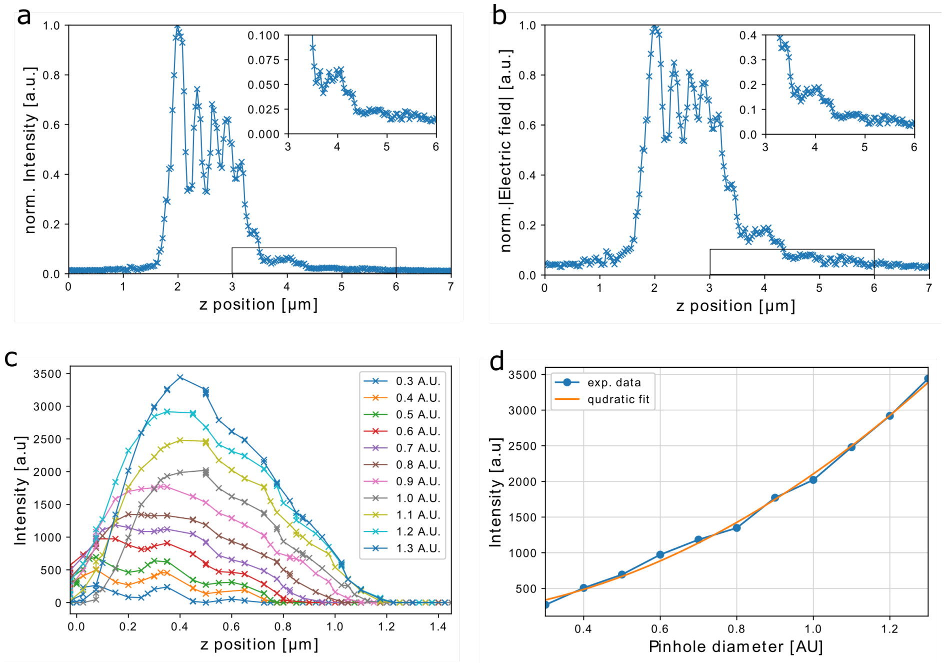

Supplementary Figure 1: Measurement of reference field intensities. **(a)** Intensity profile for 0.3 A.U. and maximum laser power settings as used e.g. for nucleus stack measurement. **(b)** The square root of the intensity plotted in (a) to portray the dependency of the reference field. **(c)** Measured depth profiles of the reflected intensity of the reference field for various pinhole sizes. **(d)** Maximum intensity dependency on pinhole diameter.

Supplementary Note 2: Background correction

To assign a contrast to C-iSCAT images and thus acquire quantitative information about the sub-cellular structure and dynamics, we first have to determine the background level I_{bg}. For fields of view including regions of bare cover glass, one can readily assess the reflected intensity I_{ref}, which can serve as the background. One can also first characterize the system (see Supplementary Figure 1a) and use that pre-calibrated information.

For a more general approach, we approximate a background by applying a low-pass filter to blur the surrounding of the structure of interest. Here, we apply a Gaussian kernel of varying width and determine an empirical cut-off kernel size to maintain the visibility of the feature of interest while blurring the environment. To choose the width of the Gaussian kernel, we performed background corrections with various kernel sizes. The results are shown in Supplementary Fig. 2 and labeled with the size of the kernel in units of the PSF width $\sigma_{\mathrm{PSF}} \simeq 150$ nm = 5 px. We see that choosing the kernel smaller than $8\sigma_{\mathrm{PSF}}$ results in structural features being subtracted as part of background. A kernel between $16\sigma_{\mathrm{PSF}}$ and $32\sigma_{\mathrm{PSF}}$ works well and was hence used for the background estimation of our data sets.

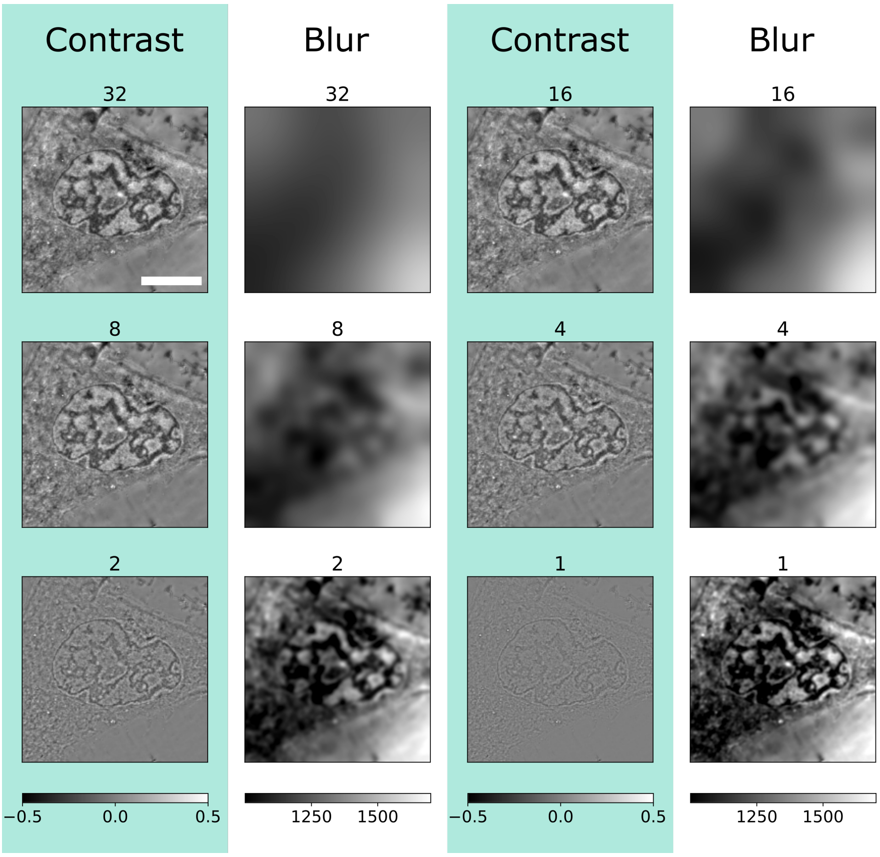

Supplementary Figure 2: Background estimation with various Gaussian kernel sizes. The size of the kernel is given in the units of PSF width $\sigma_{\mathrm{PSF}} \simeq 150$ nm = 5 px. Color scale for contrast goes from -0.5 to 0.5. Color scale for the background goes from 1000 to 1700 pixel counts. Scale bar is 10 µm.

Supplementary Note 3: PSF characterization

The point spread function (PSF) of wide-field iSCAT has been treated in depth in a previous publication (1). A quantitative analysis of the PSF in confocal iSCAT is more complex because 1) the pinhole function has to be properly implemented, especially for very small pinholes and 2) the wavefronts of the reflected field are modified as the focus is changed in the common-path mode. A theoretical treatment will be presented in a future article. In the current work, we have characterized the PSF experimentally. The result for a nanoparticle on the cover glass are shown in Supplementary Fig. 1 of the main manuscript.

To characterize the PSF at larger depths above the cover glass, we produced a model 3D sample. Here, we first immobilized 100 nm fluorescent polysterene beads (λ_{ex}= 505 nm; λ_{em}= 515 nm) on a cover glass coated with Poly-L-Lysine. Next, we added sparsely distributed a different set of beads (λ_{ex}= 580 nm; λ_{em}=605 nm) in a 3D matrix of glycergel. We measured a FoV of 15 µm laterally, a depth of 4 µm and a voxel size of 30 nm in each dimension. Supplementary Figure 3a shows a 3D representation of the beads on the coverglass (green) and those in the depth of the sample (orange).

Supplementary Figure 3b presents confocal iSCAT and confocal fluorescent axial PSFs for several examples of nanoparticles located at various depths inside the sample. The PSFs were obtained by averaging PSF of multiple beads in 30 nm bins. Two important observations can be made: 1) the axial profile of the C-iSCAT PSF experiences considerable changes at different heights, 2) the correspondence between the centers of the fluorescence and C-iSCAT PSFs is nontrivial. The first effect stems from spherical aberrations (see next section) and is similar to the observations in wide-field iSCAT (1). The second effect is caused by axial chromatic aberration.

To scrutinize the effect of chromatic aberrations, we axially localized a large number of nanoparticle using their fluorescence images. In Supplementary Fig. 4a, we plot the C-iSCAT central contrast of each particle (vertical axis) as a function of the particle position determined from its fluorescence signal (horizontal axis). The deviation of the iSCAT data from the diagonal dashed line can be explained by the axial chromatic aberration. The plot in Supplementary Fig. 4b displays a close-up of a smaller region in (a).

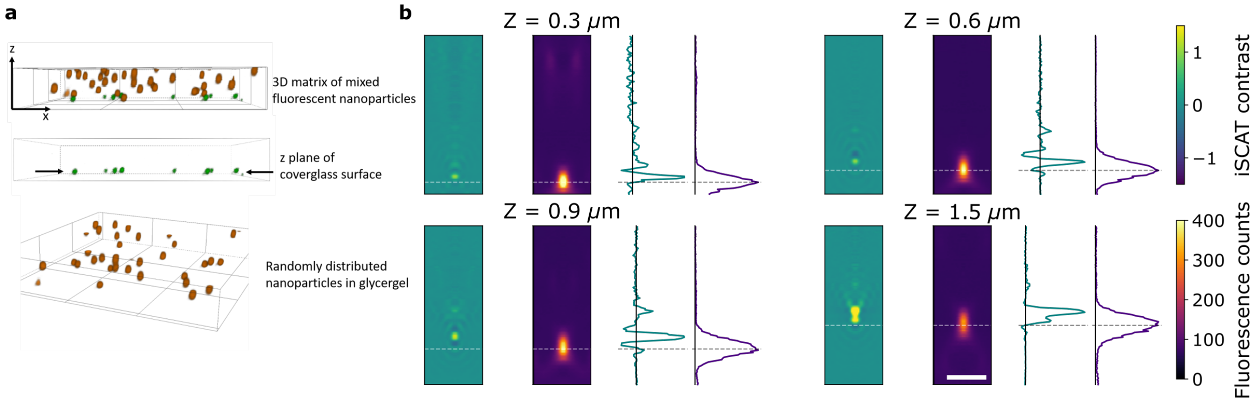

Supplementary Figure 3: Measurement of the experimental confocal iPSF. (a) 3D reconstruction of the scanned volume containing the nanoparticles at the surface (green) and the randomly distributed nanoparticles (orange) in space. **(b)** Examples of C-iSCAT and fluorescence PSFs for particles located at different z-positions. Next to each pair of plots a central line cut of the PSF is plotted for C-iSCAT (teal) and fluorescence (purple) signals. Horizontal dashed line shows the particle position as extracted from fluorescence. Scale bar is shown in the bottom right plot and is the same for all plots: 1 µm

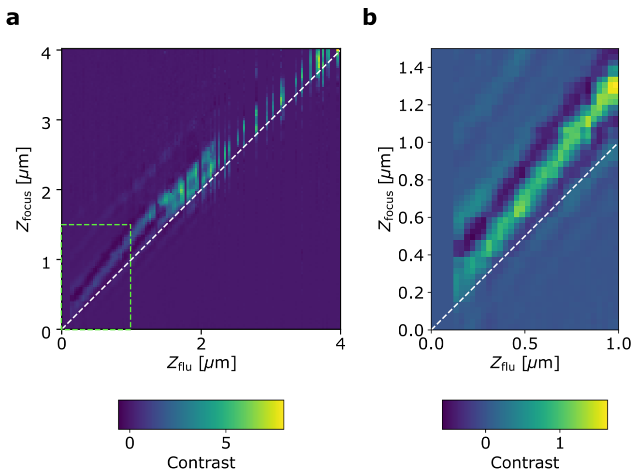

Supplementary Figure 4: Experimental iPSF characterization. (a) A two-dimensional (2D) map of the contrast of the centroid of the iPSFs as a function of both the position of the simultaneously recorded fluorescence PSF and the defocusing of the system. The dashed white line is a line with slope of 1. Some bins are empty because we found no particles at those heights in the scanned region. **(b)** Zoom-in on the region marked with a dashed green rectangle in (a).

Supplementary Note 4: Spherical aberration

Spherical aberration (SA) is a ubiquitous phenomenon in most imaging systems (2; 3). In commercial objectives, SA is usually best corrected for the interface between the cover glass and the sample (water). At more depth SA typically becomes more pronounced. Some objectives have a correction collar for partial compensation of the effects of SA. Another workaround employs water or silicone immersion objectives in order to match the sample's refractive index. In a previous publication, we showed that SA results in a shift and asymmetry of the PSF in W-iSCAT (4).

To examine the effect of SA on imaging extended surfaces, we measured the cleaved end of an optical fiber as a well-defined interface at different heights. The fiber had a diameter of 120 µm and was attached to a piezo-driven translational stage for accurate z-positioning. Supplementary Figure 5a shows the measurement scheme. At each given z-position of the fiber end, we recorded z-stacks over a range of $\pm 4\mu$m. An example of a yz image of the fiber end is shown in Supplementary Fig. 5b for the upper half space. The oscillatory behavior is a typical signature of SA, which has been documented in the literature (2; 3). Supplementary Figures 5c-f display z-cuts from measurements performed at different heights for both oil and water immersion objectives. We note that the signal represents a simple reflection intensity because the effect of interference is negligible as the reflected light is strongly masked by the pinhole for these heights. Furthermore, it is worth noting that a larger fraction of light that is reflected from an extended surface makes it through the pinhole than its counterpart scattered by a nanoparticle. Therefore, the visibility of the oscillations drops more slowly in the former case than in the latter.

Supplementary Note 5: Scan speed

The imaging speed of the confocal scanner is limited by the galvo mirror and depends on the number of scanned positions, i.e., the effective pixel size and number. We attach a matrix of FOV versus achievable frame rate that shows the technical limitations of the confocal scanner. Confocal iSCAT is a shot-noise limited technique, in that there is no upper limit of the number of photons that can contribute to the detected intensity. In other words, as opposed to the signal in fluorescence microscopy, which is ultimately limited by the emission rate of fluorophores, the scattering signal can be continuously increased as long as

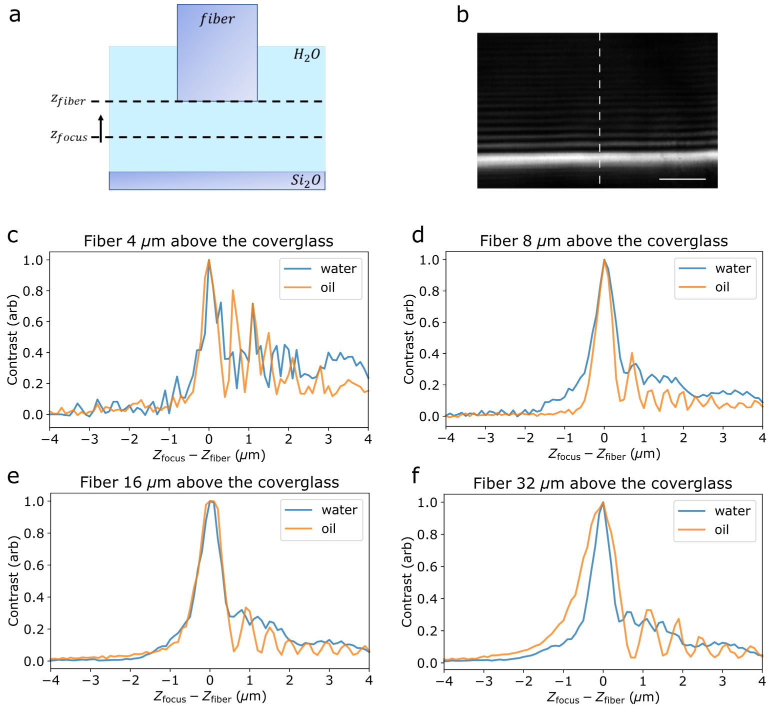

Supplementary Figure 5: Experimental characterization of spherical aberration. (a) Measurement schematic of cleaved fiber tip at different z positions above the coverglass. For each fiber position a z-stack of ±4 µm around the tip position was recorded. **(b)** Exemplary YZ image of the fiber tip. Scale bar is 3 µm **(c-f)** Measured intensity profiles of fiber tip at 4, 8, 16 and 32 µm above the coverglass. Measurements were carried out with an oil immersion objective (orange) and a water immersion objective (blue).

one avoids the optical damage threshold of the sample (see section 12). Since commercial CLSMs[1] are mainly used for fluorescence microscopy with a limited photon budget, the speed limitations of the galvo mirror are not prohibitive. iSCAT measurements, however, could benefit from faster scanning technologies.

	Galvano scanner	Resonant scanner
1D scanning	5200 lines per second	15600 lines per second
2D scanning	130 fps (512x32 pixels)	420 fps (512x32 pixels)
Full frame scanning	10 fps (512x512 pixels)	30 fps (512x512)

Table 1: Matrix of various FoV sizes and resulting scan speed with galvano scanner and resonant scanner.

To address the technical imaging speed limitation in confocal iSCAT imaging, we have added a customized WF-iSCAT setup to the microscope stand. This combination allows high-speed single particle tracking in the range of 10 kHz while simultaneously imaging cellular structures with high spatial resolution in confocal iSCAT.

Supplementary Note 6: Multiplane reconstruction workflow

A rigorous approach to 3D localization of nano-objects would involve the de-convolution of the measured signal and the expected 3D response function such as a PSF. However, the axially extended and oscillatory nature of the PSF in C-iSCAT makes it very challenging to scan a super-wavelength volume with a diffraction-limited resolution because the measured signal at a given voxel can be affected by the nontrivial contrast

[1] https://www.microscope.healthcare.nikon.com/about/news/nikon-releases-confocal-microscope-with-worlds-largest-field-of-view

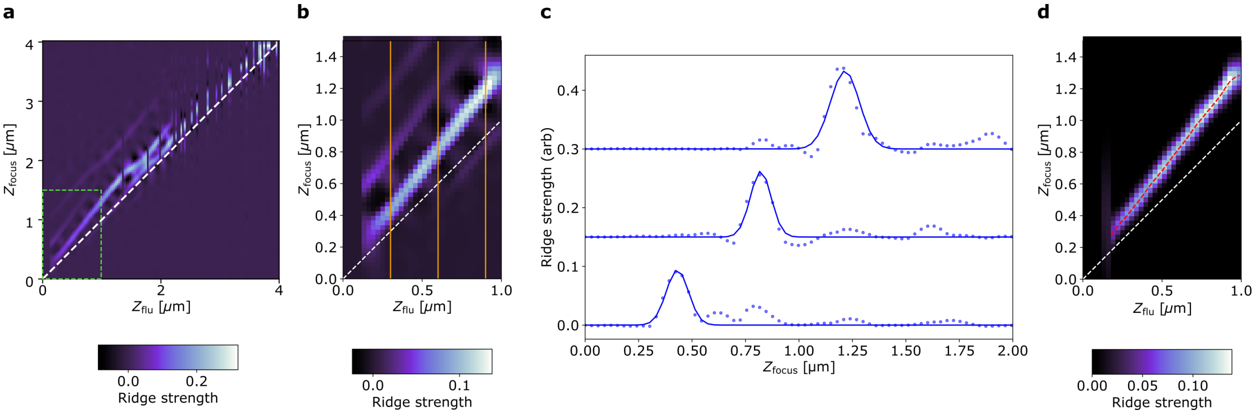

Supplementary Figure 6: PSF localization using a ridge filter. **(a)** Result of a ridge filter applied to Supplementary Fig. 4a. The dashed white line is a line with slope of 1. **(b)** Close-up of the region marked with a dashed green rectangle in (a).**(c)** Examples of several linecuts indicated with vertical orange lines in panel (b) as well as the fits to a Gaussian function. **(d)** 2D map of all fit functions. Dashed red line indicates the extracted position z_0.

reversal of an axially neighboring region. However, we have developed a scheme for high-resolution mapping of the 3D *surface contour* of an extended object such as the nucleus. Here, we describe our procedure.

First a z-stack of the desired structure is recorded with a range larger than the axial FWHM of the confocal iPSF shown in Supplementary Fig. 1 of the main paper. Each recorded focal position is background corrected by defining a background in each plane according to Supplementary Fig. 2 and normalizing the detected intensity stack to it. In some cases, it might be useful to segment the target structure in each plane. To do that, we have used a cGAN to allow for label-free detection of target structures within confocal iSCAT data sets. This provides an alternative to the molecular specificity that is usually provided by fluorescence (see below).

6.1 Extracting a ridge

As seen in Supplementary Fig. 5b, the reflection signal from an interface can contain an oscillatory behavior, but the largest fraction of power is found in the first lobe. It is, thus, sound to apply the well-established algorithms of ridge detection (5; 6) to the XZ and YZ C-iSCAT images of an interface. After application of the ridge detection algorithm, we we pick the local maximum corresponding to the ridge of interest (usually most prominent) and automatically follow it outwards on the extended structure.

Supplementary Figure 6a shows the result of the application of the ridge filter to the data from nanoparticles presented above. A ridge filter includes smoothening the image with a Gaussian kernel (chosen to match the width of the ridges to detect, here $\sigma = 2$ px), calculating the Hessian matrix for each pixel and then determining its eigenvalues. Positive eigenvalue indicates dark lines on light background, while negative eigenvalue indicates bright lines on dark background. When we detect bright lines, we always multiply the eigenvalues by -1 so that each ridge corresponds to a local maximum in the ridge filter image. Supplementary Figure 6b shows close-up of Supplementary Fig. 6a, which is indicated with a dashed rectangle. Supplementary Figure 6c displays several line cuts shown in Supplementary Fig. 6b, at the position indicated by orange lines as well as Gaussian fits to those line cuts. Finally, Supplementary Fig. 6d shows the 2D map of the fit functions.

6.2 Fitting a model function based on the C-iSCAT PSF for sub-pixel localization

An alternative to detecting the first axial maximum of the reflection signal is to locate the interface of nano-object by fitting its signal to an empirically assessed model function. As shown in Supplementary Supplementary Figs. 3 and 5, the exact form of the response function depends on the imaging depth. While

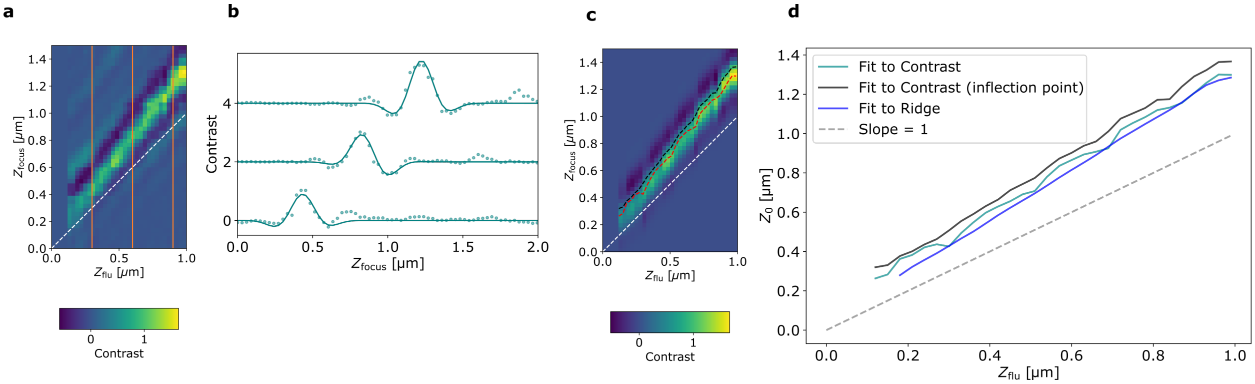

Supplementary Figure 7: PSF localization by fitting an oscillatory function. **(a)** Same as Supplementary Fig. 4b, re-plotted for convenience. The dashed white line is a line with slope of 1. **(b)** 2D map of all functions fitted to data in (a). Dashed red line indicates the extracted position z_0. Dashed black line indicates the inflection point. **(c)** Examples of several line cuts indicated with vertical orange lines in panel (a) as well as the fits to the oscillatory function with parameters indicated in the text. **(d)** The value z_0 extracted from fitting an oscillatory function to iSCAT contrast (teal), position of the main inflection point (black) and the value z_0 extracted from fitting a Gaussian to the extracted ridge (blue) plotted versus the value of particle position extracted from fluorescence data.

a quantitative model of this function is currently not available, one can fit the detected intensity with a phenomenological function that apodizes an oscillatory behavior with a Gaussian envelope.

$$I = A \sin\left(\frac{z - z_0}{\zeta} + \phi\right) \exp\left(\frac{(z - z_0)^2}{2\sigma^2}\right). \quad (1)$$

To demonstrate the reliability of this procedure, we apply it to the nanoparticle data presented in Supplementary Fig. 4. In Supplementary Fig. 7a we re-plot Supplementary Fig. 4b for convenience. We fit the linecuts indicated by orange lines in Supplementary Fig. 7a with the function described in Eq. 1 and with σ and ζ set to 120 nm and 80 nm correspondingly. The data and the fits are shown in Supplementary Fig. 7b. In Supplementary Fig. 7c, we show the resulting 2D image of fits to all the line cuts. We show the extracted z_0 and the inflection point of the largest bright to dark transition with red and black dashed lines, respectively. Supplementary Figure 7d compares the results of the different approaches.

We note that the advantage of our analysis scheme is that no *a priori* knowledge of the refractive indices is needed.

Supplementary Note 7: Mapping a model interface

7.1 Movable cleaved fiber end

To test our interface localization method further, we used a cleaved optical fiber, which we mounted on the setup as presented earlier in Supplementary Fig. 5a. To set the stage, we show the unprocessed and iSCAT contrast images of the fiber as XY and XZ slices in Supplementary Fig. 8. Similar to the apical nuclear membrane (Supplementary Fig. 2 of the main manuscript), the fiber interface is weakly visible in the raw XZ image and very prominent in the contrast XZ image. In Supplementary Fig. 8c we show the result of application of ridge filter with width of 5 px to iSCAT contrast images. In Supplementary Fig. 8d we show the extracted fiber interface with red. To test the performance of this process, we moved the fiber by about 60 nm and 120 nm in the axial direction and localized the fiber end facet in each case.

In Supplementary Fig. 9a-d we show the processing steps. First, we localize the interface following a local maximum with a step of 2 px (100 nm) (panel a). The algorithm fails punctually at locations that contain dirt or defects (unfilled regions). We then segment the fiber and interpolate the unfilled regions

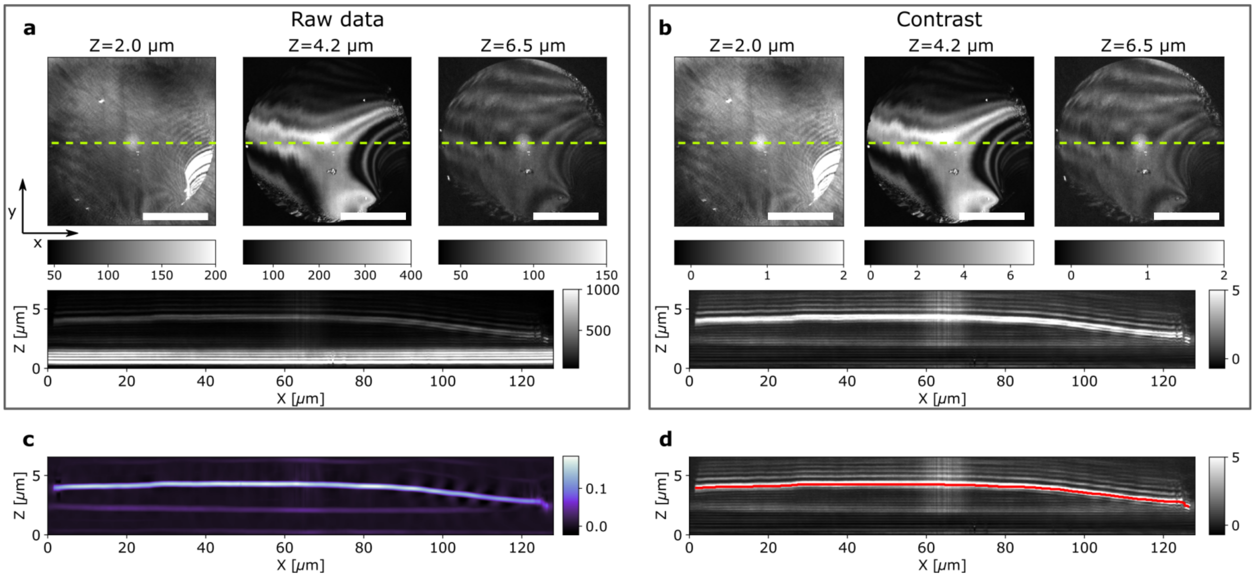

Supplementary Figure 8: Fiber interface extraction. **(a)** Raw data showing the XY (top) and XZ cuts through the fiber. Scale bar is 50 µm . The position of the cut is shown with a dashed green line. **(b)** Same as (a) but for iSCAT contrast. **(c)** Application of the ridge filter with $\sigma = 5$ px **(d)** iSCAT contrast image with detected ridge indicated with a red line.

(panel b). Next, we fit the ridge present in each line cut with a Gaussian (panel c). Here, the unfilled regions correspond to the line cuts in which the fit error (panel d) was larger than 10 nm.

Line plots of a region indicated with a blue line in Supplementary Fig. 9c are shown in panel (e) with solid lines for three axial fiber positions. The dashed curves represent the two curves at 60 nm and 120 nm offset to overlay with the 0 nm curve. The actual positions of the fiber tip deviates from the target positions mostly due to drift of the fiber tip mount. However, for extracting the surface profile of the cleaved fiber the absolute position is irrelevant. The data demonstrate that the profiles agree very well, verifying that the surface reconstruction procedure works reproducibly and robustly. In Supplementary Fig. 9f we show the difference between 60 nm and 0 nm curve as well as 120 nm and 0 nm curve. The legends report the resulting mean and standard deviation of the difference in each case.

7.2 Nanofabricated staircase sample

To realize a model system for interfaces close to the cover glass, we also fabricated and imaged a nanoscopic staircase structure. Here, we used repetitive chemical etching of a borosilicate glass substrate that was coated with a thin layer of TiO2 to generate steps of 100 nm and 150 nm depth. The interface between glass and TiO2 serves as a reference field to allow for measuring the varying height of the steps. We measured the structure with 30 nm voxel size and a total number of 96 focal planes. Supplementary Figure 10a-c shows in-focus and out-of focus planes in C-iSCAT. The corresponding height extraction is displayed in Supplementary Fig. 10d with a 2D representation of the fit error in Supplementary Fig. 10e. Here, it was not necessary to apply ridge detection, but a simple constant initial guess of z_0 in our model function was sufficient. For fitting the model function we subtracted the background from the detected intensity without normalizing it. In order to have ground truth data of this structure, we measured the same region with an AFM. The resulting 3D topography and raw data are shown in Supplementary Figs. 10e and 10g, respectively.

For comparison, we also generated line profiles both in C-iSCAT and corresponding AFM data set, which are overlayed in Supplementary Fig. 10h. Supplementary Figure 10e is a 2D representation of the fit error in order to assess the localization precision with a mean value along the indicated line of 8.4 nm. For estimating a lower limit to the localization precision we measured the standard deviation along the indicated white line both in AFM and iSCAT. The mean value of the standard deviation gives a lower limit of 2.5 nm for AFM and 3.5 nm in iSCAT.

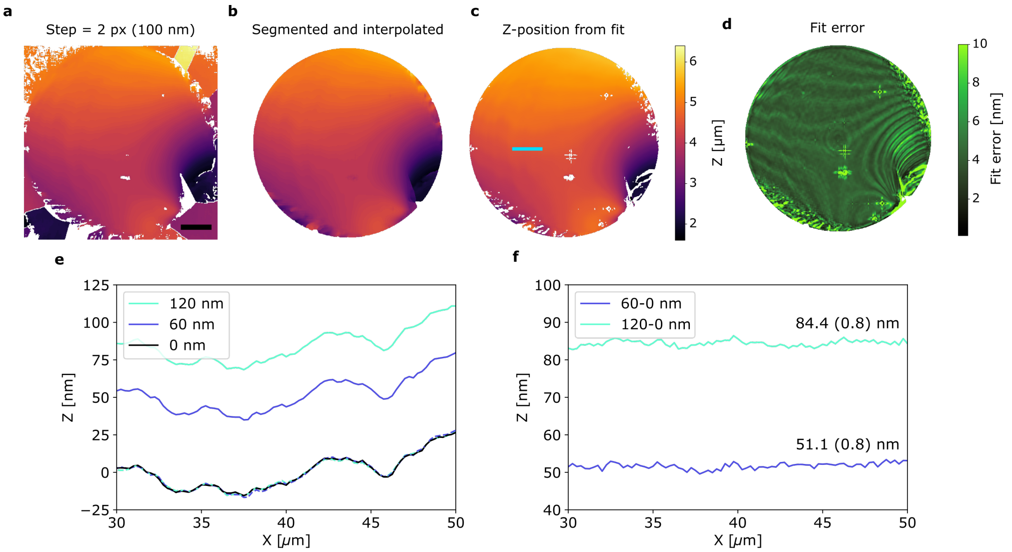

Supplementary Figure 9: Details of fiber interface extraction and measurement of the fiber at several Z-positions. **(a)** Fiber interface extracted by selecting a point on the ridge in the center and finding nearest local maxima of the neighbors moving radially outwards. Maximum allowed step between ridge locations in two neighboring pixels is 2 px (60 nm). Scale bar is 20 μm. **(b)** The image for (a) segmented and interpolated using nearest neighbor interpolation. This image is used as the initial guess for fiber position. **(c)** Extracted Z-position from the fit of a Gaussian function to the ridge **(d)** Fit error on the fiber position **(e)** The solid lines show the cuts along the the green line in panel (c) for fiber located at different relative heights above the coverglass as read out from the piezo. The dashed lines show the same profiles but displaced to overlap. **(f)** The difference of curves plotted in (e). The numbers indicate the mean and standard deviation of the difference.

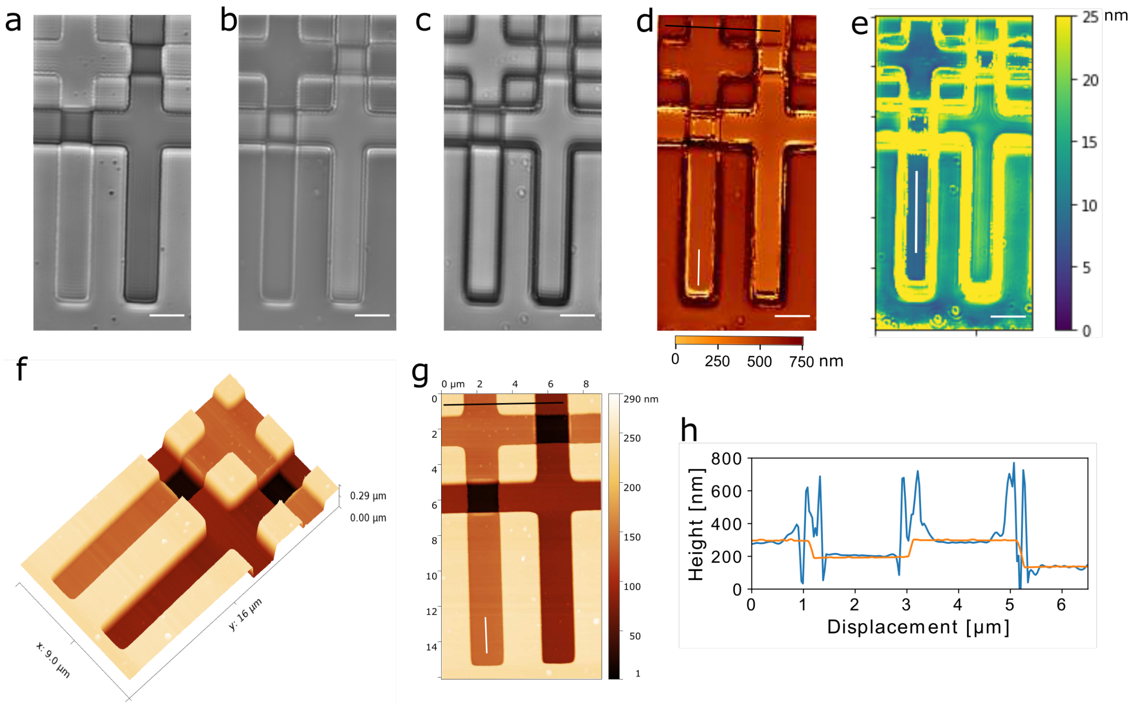

Supplementary Figure 10: Confocal iSCAT imaging and height extraction of staircase sample. **(a-c)** In-focus and out-of-focus confocal iSCAT images of two crossing steps. Scale bar 2 µm. **(d)** Extracted height profile based on multiplane reconstruction. **(e)** 2D representation of the fit error. Mean value along the white line in one step is 8.4nm. AFM measurement of TiO2 surface as **(f)** 3D surface plot and **(g)** height map.**(h)** Measured height profiles with confocal iSCAT (blue) and AFM (orange) along indicated black line in d) and g).

Supplementary Note 8: Mapping the nuclear envelope

Having explained the procedure for multiplane reconstruction of interfaces in model systems, we now demonstrate it for the case of the nuclear envelope presented in Supplementary Fig. 2. First, one records a z-stack from the part of the cell that includes the nucleus. Examples of the individual frames from the z-stack are shown in Supplementary Fig. 11a (top row). The nucleus is clearly visible, especially in the first image recorded close to the basal nuclear membrane. If we plot a YZ cut along the dashed yellow line through the raw data (see Supplementary Fig. 11a, bottom row), we see that the signal is dominated by the reflection from the cover glass. In Supplementary Fig. 11b we show the iSCAT contrast, calculated as described in section 2 of the SI using a Gaussian with a kernel of 101 px ($\approx 20\sigma_{\mathrm{PSF}}$). While the contrast XY images shown in the top row do not look qualitatively different from the raw XY images, the YZ cut shows previously masked features: basal and apical nuclear envelopes become very pronounced. The basal membrane appears as a single contrast inversion, while the apical membrane has a more complex structure caused by spherical aberration. We thus localize the lowest ridge as a good approximation to the actual position of the membrane (see section 7.1 of the SI).

Several approaches are possible here ranging from manual annotation to using a deep neural network. We decided to use an automatic approach with some manual input. We first apply a ridge filter, then choose the point on a prominent ridge and reconstruct the whole ridge from this point automatically. The outcome is shown by a red curve in Supplementary Fig. 11d, showing a very good agreement with the shape of the nucleus obtained from confocal fluorescence imaging (see Supplementary Fig. 11e), extracted by fitting a Gaussian to individual linecuts and applying a median filter with kernel size = 3 px to remove the outliers.

Supplementary Figure 12a shows the results when a point in the center of the nucleus is selected and the local maximum on the basal ridge was followed from this point outwards. To prevent the algorithm from "jumping" between adjacent local maxima, we require that the position of the maximum can change by less

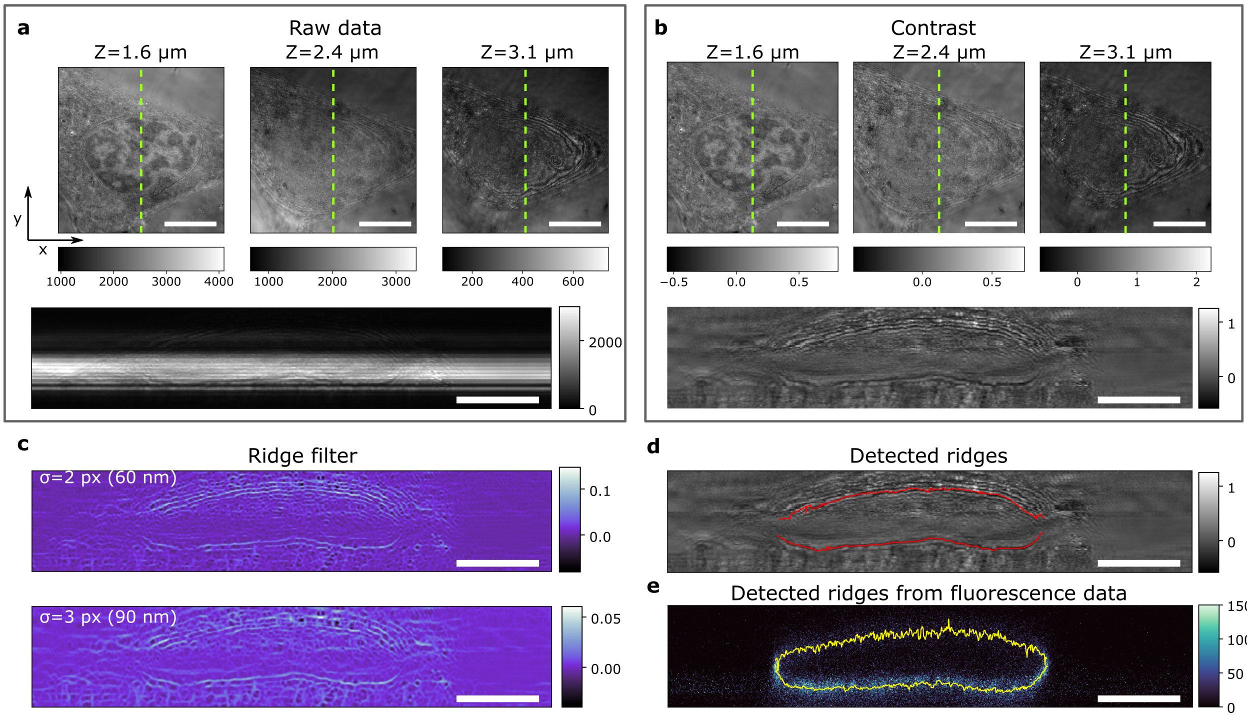

Supplementary Figure 11: Nuclear membrane extraction (a) Raw data for the XY (top) and YZ (bottom) cuts through the nucleus along the dashed green line. Top: scale bar is 10 µm, bottom: scale bar is 5 µm. **(b)** Same as (a) but for iSCAT contrast. **(c)** Application of the ridge filter with $\sigma = 2$ px (top) and $\sigma = 3$ px (bottom). Scale bar is 5 µm. **(d)** iSCAT contrast image with detected ridges indicated with a red line. **(e)** Fluorescence YZ cut and a reconstructed YZ profile from fluorescence data (yellow line).

than 2 px in a single step. The difference is calculated from the median of 5x5 pixels present in the vicinity of the pixel of interest. It is clear that the nucleus is well detected. For the region outside of the nucleus, where no ridge is present, the algorithm detects noise. In the next step, we run the same algorithm on the image that was yielded from the first step, shown in Supplementary Fig. 12a. Moreover, we allow the position of the local maximum to change by 4 px in a given step and then apply nearest neighbor interpolation over the few remaining empty spots. The outcome is shown in Supplementary Fig. 12b. To achieve a better result, we repeat the procedure, but starting from different points on the ridge (up to 10 different points). The results are averaged together and segmented by using a random forest classifier (7; 8) on the image in Supplementary Fig. 12b (see Supplementary Fig. 12c). In Supplementary Fig. 12d, we show the 3D plot of this outcome. As a comparison, we plot the map of the nuclear envelope extracted from fluorescence data in panel (e). Here we applied a ridge detection algorithm with $\sigma = 9$ px (270 nm) to the fluorescence XZ cuts and fitted a Gaussian to the ridge. We note that there is a clear correspondence between the outcomes in (c) and (e) as well as (h) and (j).

In the panels f-g the same procedure is applied to the apical nuclear envelope. As a further demonstration, we repeated the analysis on basal envelopes of three other HeLa cell nuclei shown in Supplementary Fig. 13. The nucleus shown in Supplementary Fig. 13a-c has an indentation on the left side, while the nuclei shown in Supplementary Fig. 13d-i both have a sizeable indentation on the bottom.

To achieve sub-pixel localization, we employ two approaches: 1) by fitting a Gaussian function to the detected ridge itself, as described in section 6.1 and 2) by fitting an oscillatory function based on the empirical iPSF data to individual XY-Z line cuts in iSCAT contrast, as described in section 6.2. In both cases the pixel-precise position of the ridge is taken as an initial guess. For fitting a Gaussian to the ridge all parameters are kept free, and the half-width of the Gaussian is set to be smaller than 100 nm. The 2D plots of the XZ cuts and their fits are shown in Supplementary Fig. 14a. The individual cuts and fits along the vertical lines in Supplementary Fig. 14a are shown in Supplementary Fig. 14b. Supplementary Figure 14c shows the extracted z-position of the basal nuclear membrane.

For the fit with the oscillatory model function, the values for ζ and σ are the same as in the case of the nanoparticles. As previously described, we determined the inflection point corresponding to the maximum

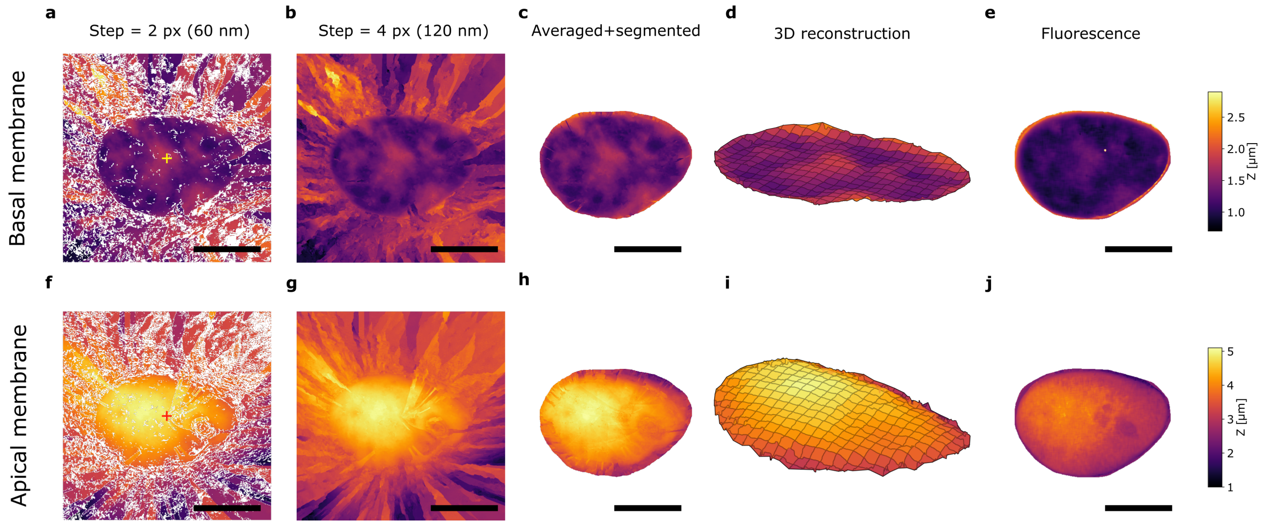

Supplementary Figure 12: Extracting a selected ridge. **(a)** Basal nuclear membrane extracted by selecting the point on the ridge in the center (yellow cross) and finding nearest local maxima of the neighbors moving radially outwards. Maximum allowed step between local maxima locations in two neighboring pixels is 2 px (60 nm). **(b)** Repeat the procedure but starting with image in (a) and allowing steps of 4 px (120 nm), interpolate the remaining empty areas with nearest neighbor interpolation. **(c)** A segmented nucleus formed as average of 10 different variants with different manually selected starting points. **(d)** Same as (c) but plotted in 3D. **(e)** The basal nuclear membrane extracted from fluorescence data. **(f-j)** Same as (a-e) but for apical membrane. Scale bar (a-j) is 10 µm.

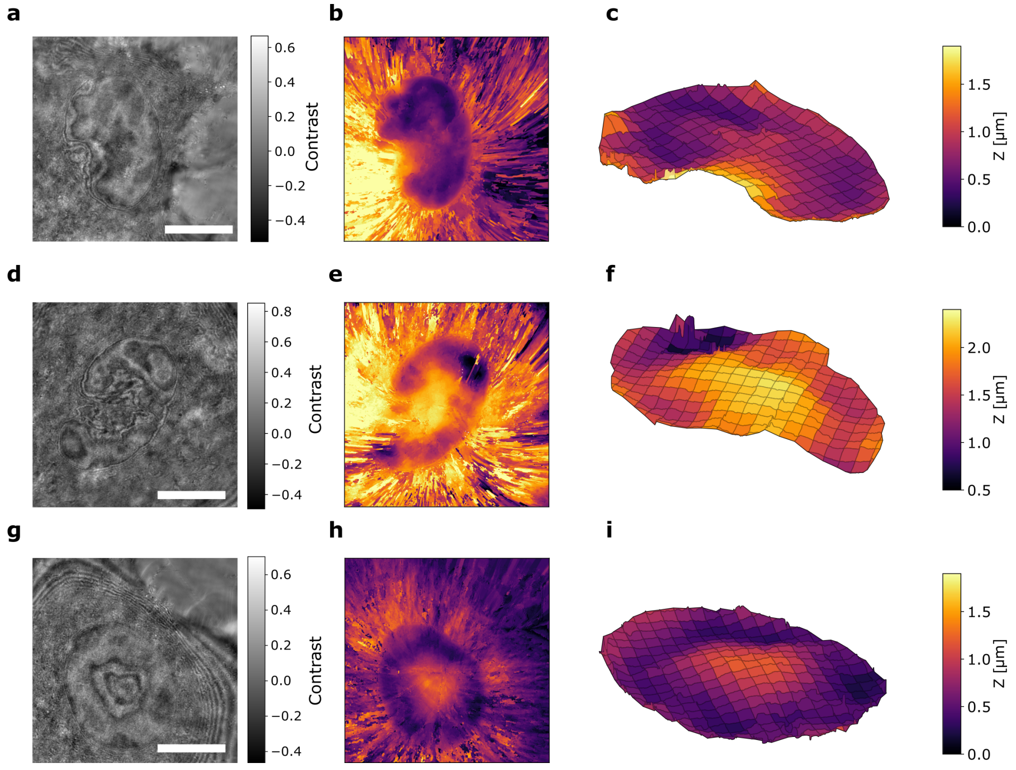

Supplementary Figure 13: Examples of basal membrane extraction for three other nuclei. (a) The iSCAT contrast image of the nucleus. Scale bar is 10 µm. **(b)** The extracted basal nuclear membrane (continuous shape) on a noisy background. **(c)** A 3D plot of segmented basal nuclear membrane **(d-i)** Same as (a-c) for two other nuclei.

bright-to-dark transition. Since the line cuts might be noisy (see Supplementary Fig. 14e), the final position is allowed to deviate from the initial guess by up to 200 nm to avoid spurious peaks. The 2D maps, individual fits and the extracted z-position are shown in Supplementary Fig. 14d-f. The results of the two methods are comparable.

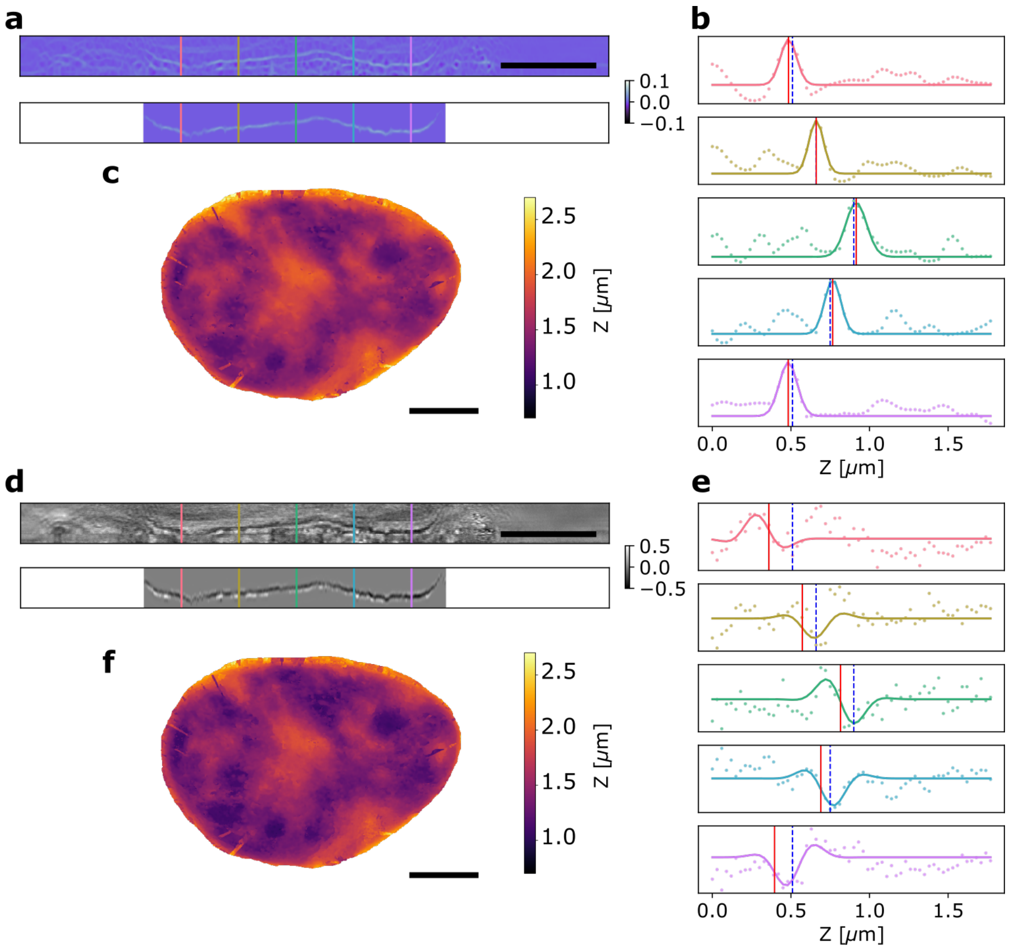

Supplementary Figure 14: Sub-pixel extraction of basal nuclear membrane. **(a)** The ridge filter image of the XZ cut of the basal nuclear membrane (top) as well as the fit to each vertical line cut with a product Gaussian. **(b)** Several linecuts along the vertical lines in panel (a) with the fits. The dashed blue line indicates the initial guess, while the red line shows the final Z-position. **(c)** The extracted Z-position. **(d-f)** Same as (a-c) but starting with a contrast image and fitting each vertical line cut with an oscillatory function. All scale bars are 5 µm

.

Supplementary Note 9: Multiplane reconstruction of the endoplasmic reticulum

We have demonstrated our multiplane reconstruction in several model systems and its application in the case of a nuclear envelope. We now apply the approach to an endoplasmic reticulum tubular network. First a z-stack with 30 nm voxel size is recorded. Supplementary Figure 15a displays a raw C-iSCAT image of a focal plane within the ER z-stack. The contrast of the ER tubules is clearly visible in the raw data. Bright and dark contrasts of that structure within one focal plane indicate the varying axial position of the ER network, which we now want to extract quantitatively. Supplementary Figure 15b shows three exemplary focal planes after background correction. In order to analyze the ER tubular network, we first segment the tubules based on the prediction of a trained cGAN (for details see section 10). We note that this step is not crucial, but it allows one to isolate the structure of interest from other features that might be present at different locations. The predicted maps for each focal plane are averaged to one map, which best represents the stationary parts of the ER network. Supplementary Figure 15c shows the resulting map which is then converted into a binary mask via Otsu thresholding. By multiplying this mask with the normalized z-stack from (b), one obtains the segmented tubular ER network as shown in Supplementary Fig. 15d. For assessing the height of the tubules, we fit the model function described in section 6.2 with fixed parameters $\sigma = 90\,\text{nm}$ and $\zeta = 60\,\text{nm}$ and a free phase. The resulting height map after this multiplane reconstruction is shown in Supplementary Fig. 15e (white dashed box in (a)) in Supplementary Fig. 15f with the corresponding 2D map of the fit error in (g). Supplementary Figure 15f depicts a close-up of (a) with the corresponding height map (g) and fit error (h). In Supplementary Fig. 15j, three exemplary voxels on ER tubules are plotted with the corresponding fit of the model function described in section 6.2. The extracted axial position is based on the inflection point of the fitted function and is marked with a vertical green line. In order to render a 3D model of the obtained height information, it is necessary to generate a new z-stack based on the height map. Here, each z position of the height map is expanded into a binary array with 1 for pixels that contain information about the structure and 0 where the structure is absent. For the 3D visualization, we assessed the median of the fit error map (Supplementary Fig. 15h) to be about 45 nm, which serves as a measure for the thickness of the structure. We then applied a 3D Gaussian kernel to the new z-stack with $\sigma = 1.5px = 45\,\text{nm}$ in each dimension. Supplementary Figure 15k shows three exemplary focal planes of the resulting new z-stack. Supplementary Figure 15l and (m) show YZ cuts along the cyan and green line as indicated in (k). A 3D rendered version of this data set based on the "3D script" software (9) is shown in the main manuscript.

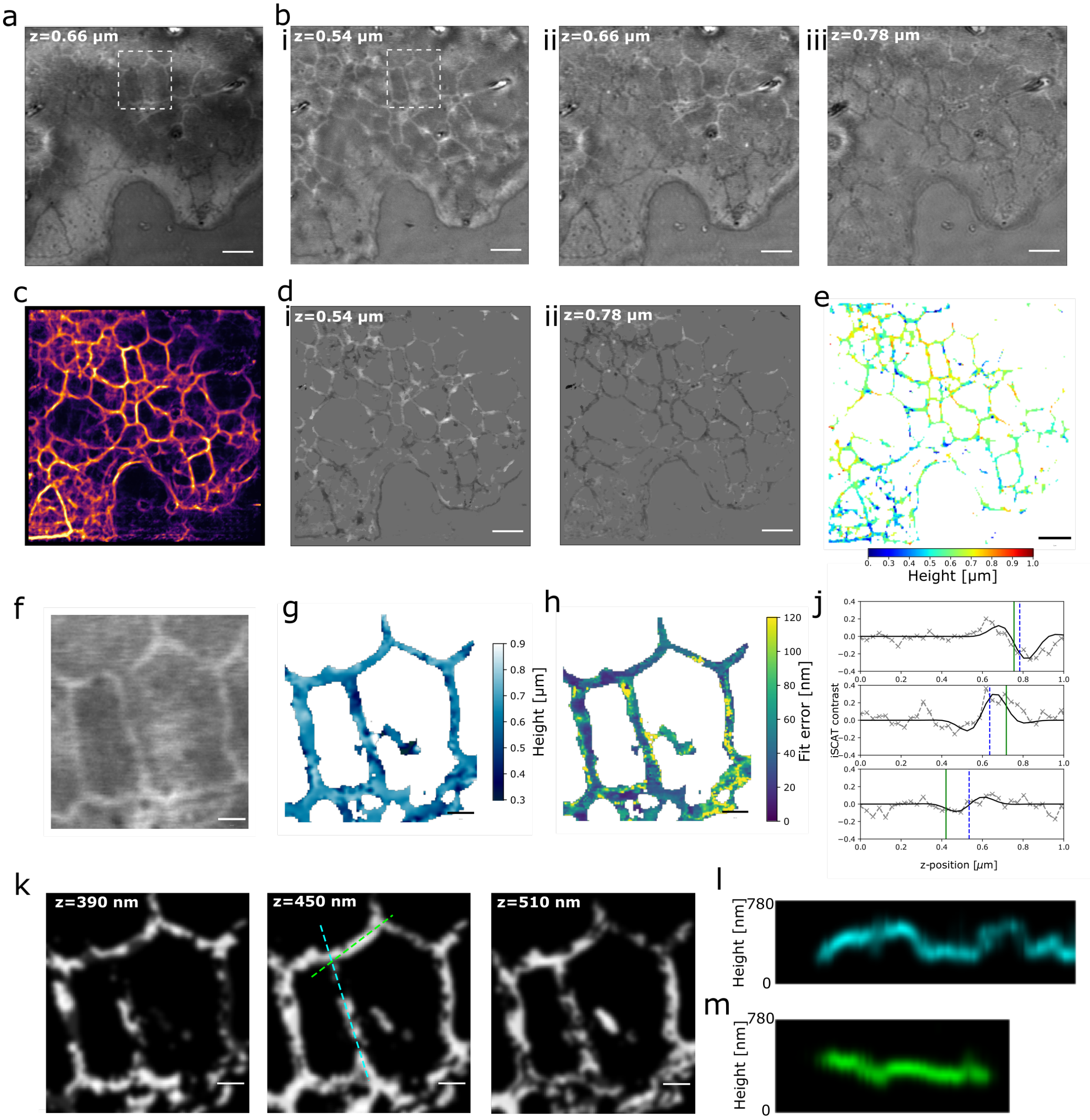

Supplementary Figure 15: 3D reconstruction of the endoplasmic reticulum. **(a)** C-iSCAT raw image of z-stack with 30nm voxel size. Scale bar 2μm. **(b)** Background corrected C-iSCAT image for three exemplary z-planes (i-iii). Scale bar 2μm. **(c)** Predicted ER-tubular network from trained cGAN (see sec. 10). Averaged intensity of all z-planes. **(d)** Segmented ER-tubular network for two z-planes shown in b). Scale bar 2μm. **(e)** Extracted height map for segmented ER tubules from c). Scale bar 2μm. **(f)** Close-up from a). Scale bar 500 nm. **(g)** Extracted height map for segmented ER tubules in the region of f). Scale bar 500 nm. **(h)** 2D map of the fit error. Scale bar 500 nm. **(j)** Exemplary voxels on ER tubules with experimental data (gray), fit with the model function (black) and resulting z position (green). **(k)** Reconstructed binary z-stack with ones where structure is present in z-plane and zero elsewhere. Three exemplary z-planes are shown after Gaussian filtering with $\sigma = 45nm$. Scale bar 500 nm. **(l)** YZ cut along the cyan line in k). **(m)** YZ cut along the green line in k).

Supplementary Note 10: Training of the cGAN neural network for segmentation

The ER in COS-7 cells is a structure of high visibility in confocal iSCAT and therefore can be segmented using simple algorithms, such as ridge detection based on eigenvalues of a Hessian matrix (5; 6), followed by a thresholding operation. However, careful fine-tuning of parameters may be needed. In order to avoid fine-tuning and to have a more general approach that can be expanded to other organelles, we trained a neural network on a set of videos in which ER was labeled fluorescently and visible in iSCAT. We used a set of 10 such videos. The size of videos ranged from 512x512 px to 1024x1024 px. Only the first few frames from each videos were used in order to have fluorescence data with sufficient SNR. This resulted in a total of 76 training images. The ER was segmented from the fluorescence images as follows: first a low-pass Gaussian filter with $\sigma = 2.5$ was applied. This was followed by ridge detection with $\sigma = 3$ and Otsu thresholding (10). Afterwards, objects with area smaller than 200 px are removed from the image to eliminate artefacts. Binary closing is applied to connect the ER network, where gaps are present due to lower fluorescence signals (8). Two examples of C-iSCAT image, segmented fluorescence (ground truth) and raw fluorescence are shown in Supplementary Fig. 16.

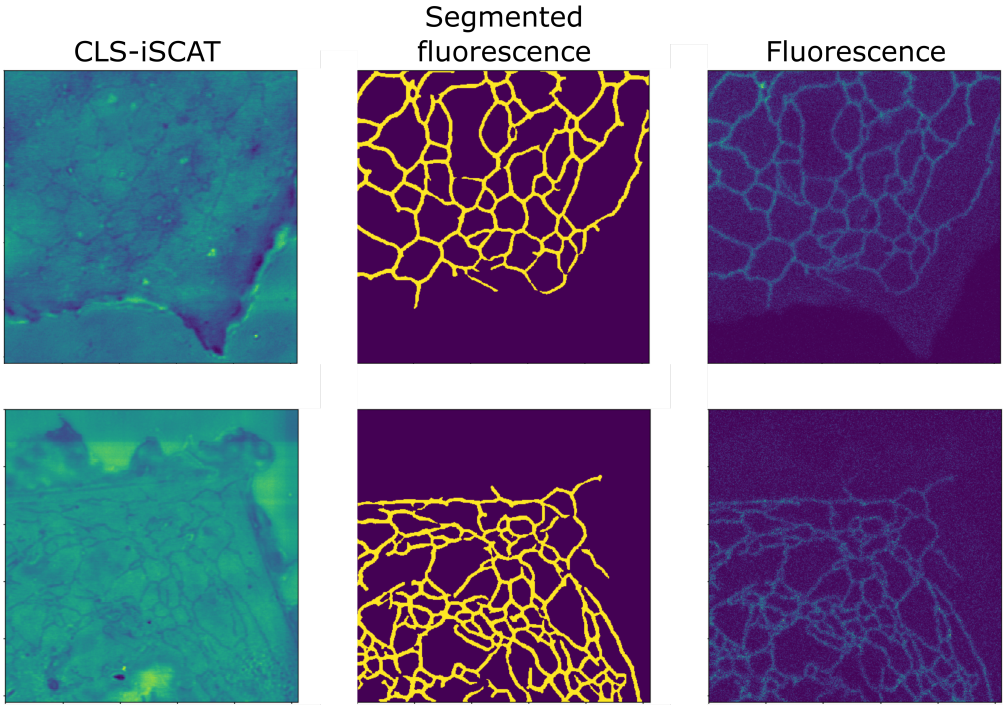

Supplementary Figure 16: Two examples of C-iSCAT images of the ER in live COS7 cells with segmented fluorescence (ground truth) and raw fluorescence used for training, respectively. All images are 512x512 px.

A Conditional Adversarial Network (11) was then trained on the data as follows. One of the 76 images was selected at random. It was rescaled along x and y by two random factors drawn from a normal distribution with width 0.1 centered around 1. A 256x256 cut-out was selected at random. Then a random rotation by 0, 90, 180 or 270 degrees and vertical mirror was applied. This was repeated 2500 times. The model was trained on this dataset over 50 epochs. The model could then be applied to 256x256 px images. The ER image shown in Fig. 3 of the main text is 256x512 px. The target image was then split into 256 overlapping images and the network was applied to each of them. The results were overlaid and averaged, yielding the image shown in Fig. 3c of the main text. This was repeated with every frame of the ER time lapse data set.

Supplementary Note 11: External reference for depth extension

As was shown in Supplementary Fig. 1, in the common-path reflection mode, the strength of the reference field is diminished as the axial positions goes away from the cover glass. In this case, the interferometric advantage is lost and the signal detected from a particle corresponds to that in a dark-field microscope. To allow for iSCAT imaging at arbitrary depths, we introduce an alternative arrangement with an external reference arm. Supplementary Figure 17 shows the schematics and picture of a compact Michelson interferometer. The setup is assembled on a baseplate that is attached to the microscope stand. To ensure that the strength of the reference beam remains constant, we have placed a second microscope objective, cover glass and water reservoir in the reference arm. A compact translation state stage can adjust the position of the cover glass ensemble to ensure that it is in the focus of the objective. The whole unit including the objective and the cover glass are mounted on a piezo-controlled translation state to control the overall length of the reference arm. This is necessary to ensure that an equal length for the sample and reference arms within the coherence length of the laser, which in our cases corresponded to about 150 µm.

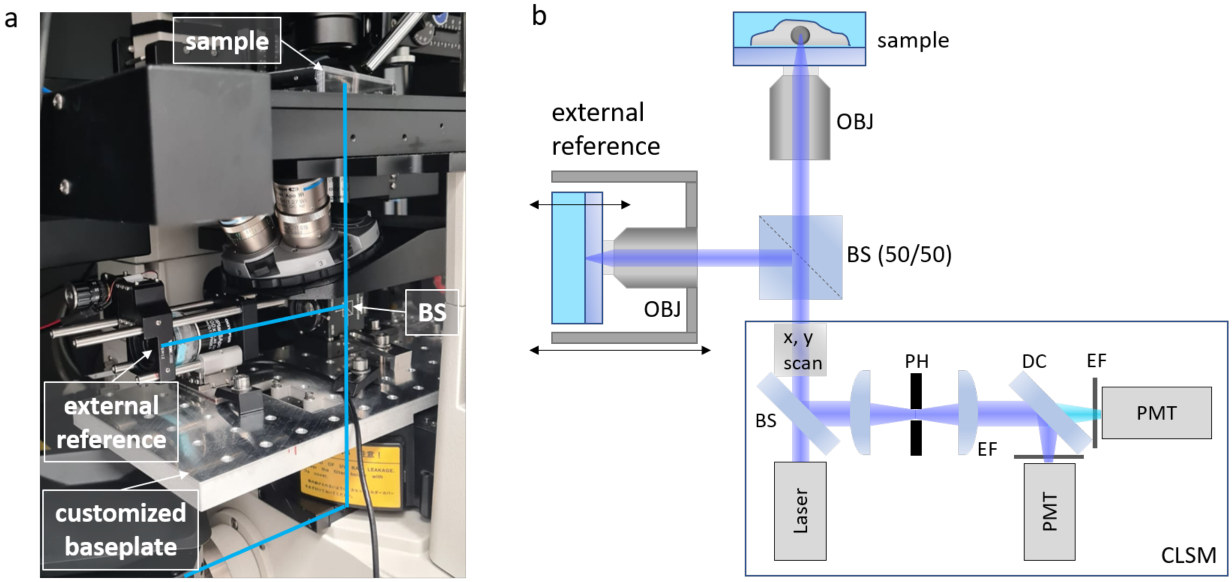

Supplementary Figure 17: Custom-made holder for external reference. **(a)** Integration into microscope stand. **(b)** Modified setup schematic showing the external reference module added to the CLSM.

To test our confocal Michelson interferometer, we used the cleaved fiber as described in section 4. Here, we kept the focal plane at a constant position of 64 µm above the cover glass and moved the fiber tip axially by $\pm 2\mu m$ around that focal position (Supplementary Fig. 18 a). Supplementary Figures 18b and 18c show the detected lateral images obtained when the fiber end was placed in the focal plane with (b) and without (c) the external reference beam. The Newton rings in Supplementary Fig. 18 are a clear evidence for interference between the two arms and demonstrate a slight tilt of the fiber tip interface compared to the external reference plane.

Supplementary Figure 18 d-g displays the axial intensity line profiles for two different pinhole sizes and two cases where the external reference arm was present and when it was blocked. The modulation of the intensity profiles in (d) and (e) provide a clear evidence for the performance of the interferometric microscope.

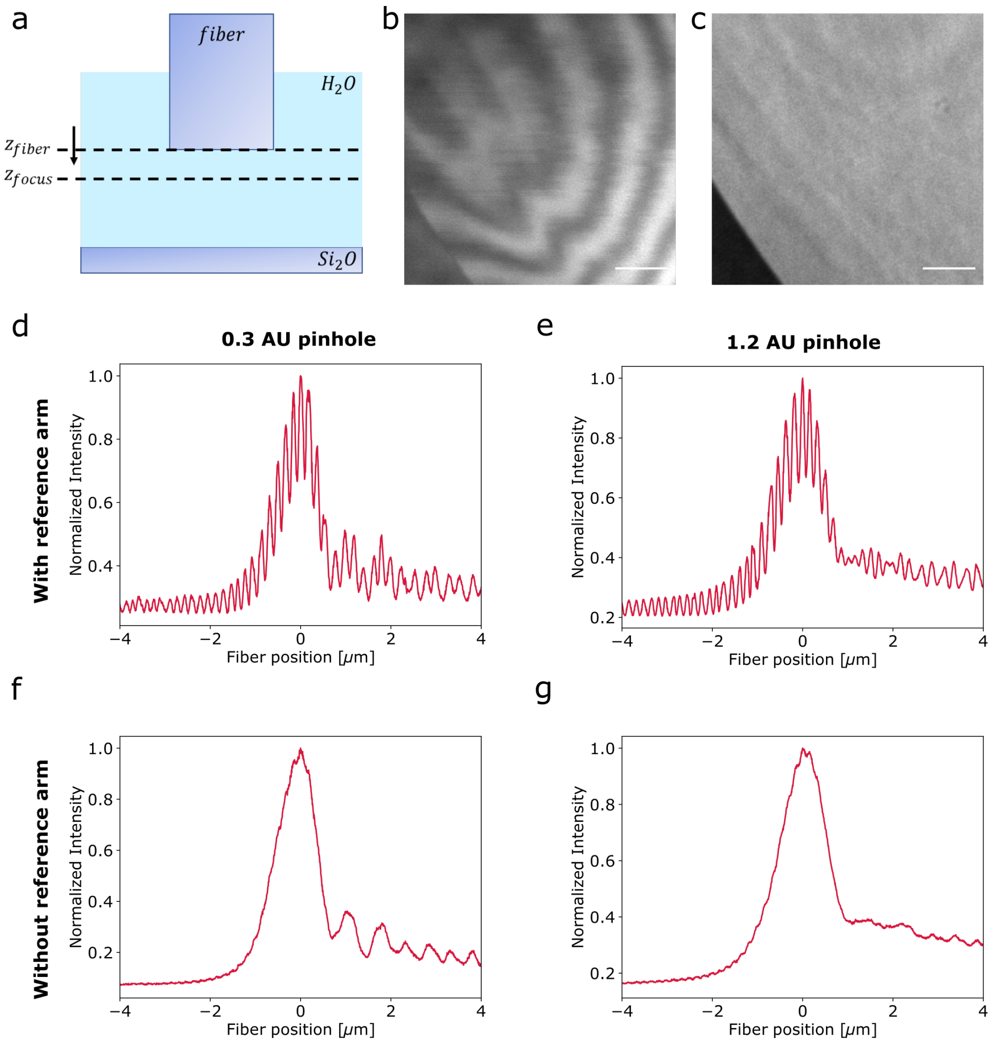

Supplementary Figure 18: External reference measurements of a cleaved fiber tip. **(a)** Measurement schematics. Lateral scans when the fiber tip was in focus at about 64 μm above the cover glass with external reference **(b)** and without **(c)**. Scale bar is 3 μm. **(d,e)** Axial intensity line profile of the fiber tip moved by ±2 μm around the focal position at a pinhole diameter of 0.3A.U. and 1.2 A.U with external reference. **(f,g)** Axial intensity line profile of the fiber tip moved for ±2 μm around the focal position at a pinhole diameter of 0.3A.U. and 1.2 A.U without external reference.

Supplementary Note 12: Phototoxicity

To assess the cell viability of our measurements, we carried out phototoxicity studies with fluorescence labelled and unlabelled COS7 cells. COS7 cells were transiently transfected with GFP-GPI using lipofectamine 3000 and imaged in Leibovitz' medium in an on-stage humidified incubation chamber at 37°C on gridded ibidi dishes to follow individual cells over time. Multiple cells in a field of view of approximately 250 x 480 µm were continuously illuminated with a laser at a wavelength of 445 nm and power of 5 µW for 240 s and a pixel dwell time of 2.2 µs (Supplementary Fig.19 a,b). After light exposure, a single image was acquired every 2 h to follow individual cells for 16 h (Supplementary Fig.19 c,d). Of 25 cells (18 fluorescent) followed over 24 h one transfected cell died (arrow), while the remaining showed no signs of apoptosis. Several cells entered mitosis (examples marked with asterisks), indicating that the exposed light dose level is generally well tolerated by both transfected and untransfected cells. Erythosin staining for apoptotic cells was negative, indicating negligible levels of phototoxicity for the used laser power settings.

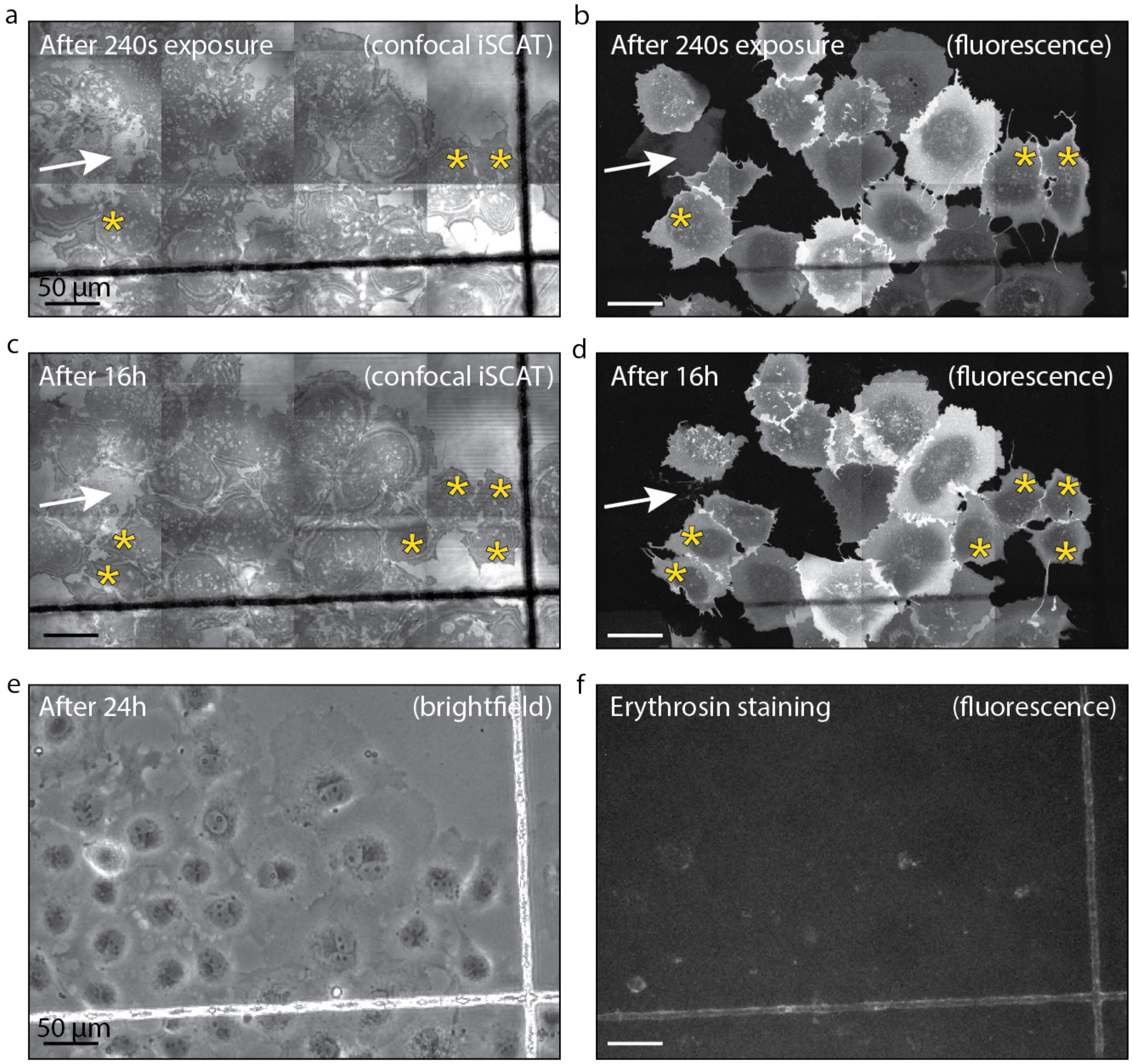

Supplementary Figure 19: Phototoxocity assessment. **(a)** COS7 cells transfected with the fluorescent membrane marker GFP-GPI were imaged for 240s in confocal iSCAT and **(b)** their fluorescence signal recorded.**(c)** After 16h cells were viable with **(d)** good fluorescence signal. One cell died (arrow) while multiple divided (asterisks). **(e)** After 24h viability was controlled in brightfield and **(f)** by staining apoptotic cells with erythrosine. Scale bars 50 µm.

References

[1] Mahmoodabadi, R. G. *et al.* Point spread function in interferometric scattering microscopy (iSCAT) part i: aberrations in defocusing and axial localization. *Optics Express* **28**, 25969 (2020).

[2] Hell, S. W. & Stelzer, E. H. K. Lens Aberrations in Confocal Fluorescence Microscopy BT - Handbook of Biological Confocal Microscopy. 347–354 (Springer US, Boston, MA, 1995).

[3] Sheppard, C. J. R. & Gu, M. Aberration compensation in confocal microscopy. *Applied Optics* **30**, 3563–3568 (1991).

[4] Gholami M., R. *et al.* Point spread function in interferometric scattering microscopy (iSCAT). Part I: aberrations in defocusing and axial localization. *Optics Express* **28**, 25969–25988 (2020).

[5] Sato, Y. *et al.* Three-dimensional multi-scale line filter for segmentation and visualization of curvilinear structures in medical images. *Med. Image Anal.* **2**, 143–168 (1998).

[6] Damon, J. Properties of ridges and cores for two-dimensional images. *Journal of Mathematical Imaging and Vision* **10**, 163–174 (1999).

[7] Breiman, L. Random forests. *Mach. Learn.* **45**, 5–32 (2001).

[8] van der Walt, S. *et al.* scikit-image: image processing in python. *PeerJ* **2**, e453 (2014).

[9] Schmid, B. *et al.* 3Dscript: animating 3D/4D microscopy data using a natural-language-based syntax. *Nat. Methods* **16**, 278–280 (2019).

[10] Otsu, N. A threshold selection method from gray-level histograms. *IEEE Transactions on Systems, Man, and Cybernetics* **9**, 62–66 (1979).

[11] Isola, P., Zhu, J.-Y., Zhou, T. & Efros, A. A. Image-to-image translation with conditional adversarial networks. In *2017 IEEE Conference on Computer Vision and Pattern Recognition (CVPR)*, 5967–5976 (2017).
