# Supplementary material for: Confocal interferometric scattering microscopy reveals 3D nanoscopic structure and dynamics in live cells
Source: Nat Commun. 2023 Apr 7;14:1962. doi: 10.1038/s41467-023-37497-7 (PMC10081331; doi:10.1038/s41467-023-37497-7)
Supplement: Supplementary file 4 — Description of Additional Supplementary Files [file 41467_2023_37497_MOESM4_ESM.docx]

**Description of Additional Supplementary Files**

Supplementary Movie 1

Description: C-iSCAT z stack through a live HeLa cell transfected with mCherry-LaminA as displayed in Fig. 2. Cell was imaged from bottom to top over ∼ 4 µm with z steps of 30 nm under physiological conditions in imaging buffer at 37 °C. Fluorescence signal from mCherry-LaminA was recorded simultaneously. Images were background corrected to display the C-iSCAT contrast. Scale bar is 5 µm

Supplementary Movie 2

Description: ER-tubules: C-iSCAT time lapse recording of a live COS-7 cell transfected with ER-EGFP as displayed in Fig. 3. Cell was imaged continuously at 4 Hz with an active focus lock system under physiological conditions in imaging buffer at 37 °C. Fluorescence signal from ER was recorded simultaneously. Images were background corrected to display the C-iSCAT contrast. Scale bar is 1 µm

Supplementary Movie 3

Description: ER-sheet: C-iSCAT time lapse recording of a live COS-7 cell transfected with ER-EGFP as displayed in Fig. 3. Cell was imaged continuously at 1 Hz with an active focus lock system under physiological conditions in imaging buffer at 37 °C. Fluorescence signal from ER was recorded simultaneously. Images were background corrected to display the C-iSCAT contrast. Scale bar is 5 µm.

Supplementary Movie 4

Description: Microtubules: C-iSCAT time lapse recording of a live COS-7 cell transfected with mEGFPtubulin and CytERM-mScarlet as displayed in Fig. 4a. Cell was imaged continuously at ∼ 1 Hz with an active focus lock system under physiological conditions in imaging buffer at 37 °C. Fluorescence signals from ER (magenta) and MTs (cyan) were recorded simultaneously. Images were background corrected to display the C-iSCAT contrast. Scale bar is 2 µm

Supplementary Movie 5

Description: Microtubules: C-iSCAT time lapse recording of a live COS-7 cell transfected with mEGFPtubulin and CytERM-mScarlet as displayed in Fig. 4a. Cell was imaged continuously at ∼ 1 Hz with an active focus lock system under physiological conditions in imaging buffer at 37 °C. Fluorescence signals from ER (magenta) and MTs (cyan) were recorded simultaneously. Images were background corrected to display the C-iSCAT contrast. Scale bar is 1 µm

Supplementary Movie 6

Description: C-iSCAT time lapse recording of a live COS-7 cell as displayed in Fig. 4f. Cell was imaged continuously at ∼ 1 Hz with an active focus lock system under physiological conditions in imaging buffer at 37 °C. Images were background corrected to display the C-iSCAT contrast. Scale bar is 5 µm

Supplementary Movie 7

Description: C-iSCAT time lapse recording of a live COS-7 cell as displayed in Fig. 4f. Cell was imaged continuously at ∼ 1 Hz with an active focus lock system under physiological conditions in imaging buffer at 37 °C. Images were background corrected to display the C-iSCAT contrast. Scale bar is 1 µm

Supplementary Movie 8

Description: C-iSCAT time lapse recording of a live COS-7 cell transfected with clathrin-mCerulean3 as displayed in Fig. 5a. Cell was imaged continuously at 1 Hz with an active focus lock system under physiological conditions in imaging buffer at 37 °C. Fluorescence signal from clathrin coated pits (cyan) was recorded simultaneously. Images were background corrected to display the C-iSCAT contrast. Scale bar is 10 µm.

Supplementary Movie 9

Description: : C-iSCAT time lapse recording of a live COS-7 cell transfected with clathrin-mCerulean3 as displayed in Fig. 5d. Cell was imaged continuously at 1 Hz with an active focus lock system under physiological conditions in imaging buffer at 37 °C. Fluorescence signal from clathrin coated pits (cyan) was recorded simultaneously. Images were background corrected to display the C-iSCAT contrast. Scale bar is 1 µm.

Supplementary Movie 10

Description: 3D representation of the basal and apical surfaces of the nuclear envelope. Color map encodes height ranging from 0 up to 4 µm according to Fig. 2 of the main paper.
